# NURSING HOME WORKER MENTAL HEALTH AND TURNOVER INTENTION AMONG PARENTS

**DOI:** 10.1093/geroni/igae098.3239

**Published:** 2024-12-31

**Authors:** Katherine Kennedy, David C Mohr, Whitney Mills

**Affiliations:** VA Providence Healthcare System, Center of Innovation in Long-Term Services and Supports, Providence, Rhode Island, United States; Veterans Health Administration, Mason, Ohio, United States; VA Providence Health Care System, VA Providence Health Care System, Rhode Island, United States

## Abstract

Nursing home (NH) staff job dissatisfaction and turnover are associated with lower care quality. However, little is known about the impact of staff mental health, parenthood, and marital status. Guided by the Job Demands-and-Resources Model (assumes positive effects of job resources and negative effects of job demands on individual and work outcomes), we compared psychological distress and job satisfaction for single parents and married parents. NH employee and manager data from Wave 4 (2011-12) of the Work Family Health Network study were combined (N=1,144) to define parents (N=682). Strata were compared on demographics, job characteristics, turnover intention, job satisfaction, and psychosocial health using bivariate tests and logistic regression models. Single parents comprised 51.8% (n=353) of the sample. In bivariate tests, single parents reported higher turnover intention and psychological distress as well as lower job satisfaction and social support than married parents (p<.001). Turnover intention was more likely endorsed when staff reported greater psychological distress (OR=1.07, 95% CI: 1.03, 1.12, p<.01). Distress was more likely with increasing work-to-family conflict (OR=1.68, 95% CI: 1.22, 2.31, p<.01) and increasing family-to-work conflict (OR= 1.78, 95% CI: 1.21, 2.63, p<.01). Distress was less likely with higher household income (OR= 0.92, 95% CI=0.87, 0.94, p<.05). Additional research is needed to understand and develop strategies to mitigate psychological distress and increase job resources among single parents to improve NH worker outcomes.

